# Barriers to Physical Activity in Low Back Pain Patients following Rehabilitation: A Secondary Analysis of a Randomized Controlled Trial

**DOI:** 10.1155/2017/6925079

**Published:** 2017-10-25

**Authors:** Andrea Schaller, Anne-Kathrin Exner, Sarah Schroeer, Vera Kleineke, Odile Sauzet

**Affiliations:** ^1^Institute of Health Promotion and Clinical Movement Science, German Sport University Cologne, Am Sportpark Muengersdorf 6, 50933 Cologne, Germany; ^2^IST-Hochschule University of Applied Sciences, Erkrather Str. 220 a-c, 40233 Duesseldorf, Germany; ^3^Department of Epidemiology and International Public Health, Bielefeld School of Public Health (BiSPH), Bielefeld University, Bielefeld, Germany; ^4^FH Münster-University of Applied Sciences, Münster School of Health, Münster, Germany; ^5^Institute of Medical Informatics, Biometry and Epidemiology, University Hospital Essen, 45147 Essen, Germany; ^6^StatBeCe, Centre for Statistics, Bielefeld University, Bielefeld, Germany

## Abstract

**Background:**

Promoting health-enhancing physical activity following rehabilitation is a well-known challenge. This study analysed the barriers to leisure time activity among low back pain patients.

**Methods:**

A subset of 192 low back pain patients who participated in a randomized controlled trial promoting physical activity was analysed. Physical activity, barriers, and sociodemographic and indication-related variables were assessed by a questionnaire. Differences in barriers between active and inactive participants were tested by Pearson's chi squared test. A logistic regression model was fitted to identify influencing factors on physical activity at six months following rehabilitation.

**Results:**

Inactive and active participants differed significantly in nine of the 19 barriers assessed. The adjusted regression model showed associations of level of education (OR = 5.366 [1.563; 18.425]; *p* value = 0.008) and fear of pain (OR = 0.612 [0.421; 0.889]; *p* value = 0.010) with physical activity. The barriers included in the model failed to show any statistically significant association after adjustment for sociodemographic factors.

**Conclusions:**

Low back pain patients especially with a low level of education and fear of pain seem to need tailored support in overcoming barriers to physical activity. This study is registered at German Clinical Trials Register (DRKS00004878).

## 1. Introduction

Low back pain exhibits a high prevalence in medical rehabilitation and high costs for social insurers in Germany [1, 2]. Physical activity and exercise are an integral part in the management and rehabilitation of low back pain [3–8]. Across all indications the promotion of health-enhancing physical activity is of utmost importance to increase the sustainability of rehabilitation [8–10]. Nevertheless, engaging in regular exercise and implementing physical activity into a daily routine are a common problem for patients following rehabilitation [11]. However, little is known about the barriers to physical activity among low back pain patients.

In general, commonly reported barriers for undertaking physical activity can be assigned to different categories:* lack of time* (e.g., due to family, household, and occupational responsibilities) [12–16],* health and quality of life* (e.g., comorbidity) [14, 17–19],* psychological barriers* (e.g., emotional or motivational problems) [15, 16, 19],* social and sociocultural barriers* (e.g., family commitments) [12, 13, 16, 18],* access issues *(e.g., financial limitations, physical environment, and lack of access to exercise opportunities) [12, 15–18],* low socioeconomic status* [12], and* lack of knowledge* (e.g., dependence on professional instruction) [12, 13, 17] or* competing priorities* [13].

In order to increase physical activity behavior among low back pain patients it is important to understand what barriers prevent them from participating. Following a research project on physical activity promotion [20] the objective of the present exploratory study was to describe the perceived barriers to physical activity among a group of low back pain patients following rehabilitation. The research questions of the present secondary analysis were as follows: (1) Which barriers to physical activity do physically inactive low back pain patients describe following rehabilitation? (2) Which barriers and sociodemographic and indication-related variables are associated with physical inactivity following rehabilitation?

## 2. Materials and Methods

### 2.1. Study Design and Data Source

The cross-sectional data analysed for this work were obtained from a subset of 192 low back pain patients who completed a questionnaire on physical activity barriers after participating in a randomized controlled trial (*T*0 = start of inpatient rehabilitation (baseline),* T*1 = six-month follow-up, and* T*2 = twelve-month follow-up).

The randomized controlled trial evaluated the effectiveness of two different approaches in physical activity promotion [20]. The intervention [20] as well as details of the study sample, including details regarding the subset of patients who completed the six-month follow-up questionnaire (*T*1) compared to the baseline sample (*T*0), [21] has already been reported elsewhere.

In brief, chronic low back pain (LBP) patients were recruited in an inpatient rehabilitation center from May 2013 until the planned sample size of 264 patients was reached in April 2014. Details of the sample size calculation are described in the study protocol [20]. The inclusion criteria were (1) age 18 to 65 years; (2) starting an inpatient medical rehabilitation treatment due to low back pain. Exclusion criteria were (1) cognitive disorders; (2) insufficient understanding of the German language; (3) any kind of surgery within the last three months; (4) posttraumatic conditions (e.g., LBP following an accident); (5) a current state pension claim. Participants were randomly assigned to the intervention group (*Movement Coaching*: comprehensive multicomponent intervention with small-group intervention, phone- and web 2.0-intervention) or the low intensity control group (two oral presentations available for download afterwards). The therapist that conducted the intervention in both study groups had a Master's degree in Sport Science with the main field of study in “Rehabilitation and Health Management.”

The main outcome was total physical activity. Written informed consent was obtained from each participant. The study was conducted in compliance with the Helsinki Declaration and was approved by the Ethics Committee of the German Sport University Cologne. The study is registered in the German Clinical Trials Register (DRKS00004878). Patients answered the baseline questionnaire (*T*0) at the beginning of the inpatient rehabilitation. The data on barriers and physical activity were collected at six-month follow-up (*T*1) by postal questionnaire.

### 2.2. Outcome Measures

Physical activity was measured with the Global Physical Activity Questionnaire [22, 23]. The GPAQ collects information on physical activity during a typical week within three areas of life (workplace, transport, and leisure time) as well as sedentary behavior. Since the differentiation of workplace and leisure time physical activity seems to be relevant in assessing health-enhancing effects in low back pain patients [24–26], we focused on leisure time physical activity solely. The GPAQ asks if physical activity during leisure time is performed during a regular week (“yes”/“no”) and subsequently measures leisure time physical activity with respect to its intensity by multiplying the minutes per week for each domain by their associated MET to create MET-min scores (the metabolic equivalent (MET) is a physiological measure expressing the expended energy of physical activities; MET is defined as the ratio of the rate of energy consumption during a specific physical activity to a reference metabolic rate). Activity specific scores are summed to create total MET-min/week (MET-min/week). Thereby, each minute of vigorous physical activity is multiplied by 8 METs and each minute of moderate physical activity by 4 METs.

To identify perceived situational barriers a patient-reported questionnaire was used [27]. Asking “how strongly do the following barriers keep you from being physically active and/or doing sports in your daily routine” the questionnaire assesses 13 situational barriers. Thereby, a situational barrier is a high risk situation which impedes the realization of the behavior change. Barriers were separated into* psychosocial and external factors* (ten items): bad weather, being tired, too much work, being in a bad mood, activities with friends, not feeling like it, wanting to stay home, TV, being depressed, and stress and* physical barriers* (three items): being ill (meaning suffering from an illness or disease or feeling unwell), pain, and injury. Beyond, we extended the questionnaire with six different* access barriers* (partner against it, sport field too far away, too expensive, difficult to organize, no sport partner, and forgot to do sport). Participants were asked to rate the perceived impact of each barrier on a 4-point Likert-type scale that ranges from 1 (not a barrier at all) to 4 (very strong barrier). Higher scores indicate that the associated barrier has a greater impact on the participant's ability to participate in sports or leisure time physical activity.

Additionally, we asked about demographic and indication-related variables by nonstandardized questions at baseline (gender, age (years), height, weight, level of education, duration of low back pain at the beginning of inpatient rehabilitation and at six-month follow-up, intensity of pain during the last four weeks, pain by physical activity).

### 2.3. Statistical Analysis

For the sample description means and standard deviation (SD) were calculated for continuous data and frequency tables (*n*; %) for categorical data. For physical activity, the number of subjects being “active” during leisure time was reported. We defined “active” reporting any physical activity during leisure time (>0 MET-min/week) and “inactive” as no physical activity during leisure time (0 MET-min/week). Low back pain at the beginning of inpatient rehabilitation (“≤12 months”/“>12 months”), level of education (“lower secondary school”/“higher level of education than lower secondary school”), and body mass index (“normal weight”/“overweight or obese”) were dichotomized.

For description of the barriers to physical activity (research question 1) we only included participants reporting to be physically inactive during leisure time (0 MET-min/week) at six-month follow-up (*T*1). The patient-oriented perception of barriers was described using frequencies.

To identify factors with physical inactivity following rehabilitation (research question 2) a logistic regression model was used. For this purpose, we dichotomized each barrier (“barrier”/“no barrier”). A barrier was considered if a participant responded with “4: very strong barrier” or “3: strong barrier”. No barrier was considered if the participant answered “1: not a barrier at all” or “2: minimal barrier”.

As we pursued an exploratory approach in our study, we first compared barriers in active and inactive participants using for each Pearson's chi squared test. Due to the explorative nature of the work we did not control for multiple testing. If there was a significant difference between the two groups, we included the specific barrier in our logistic regression model. To adjust for confounding, all sociodemographic and indication-related variables assessed were included in the model. Additionally, we controlled for the effects of the intervention from the main study by including the intervention group (“Movement Coaching”; “low intensity control group”) in the analysis. Physical activity at six months following rehabilitation (“active” versus “inactive”) was the dependent variable. Participants with missing values in dependent or independent variables were excluded. Analyses were performed using SPSS 24. The *α*-level of significance was set at *p* value < 0.05.

## 3. Results

### 3.1. Study Sample

Consort flow chart of the main study was already published [21]. The demographic and indication-related characteristics of the 192 participants in the present evaluation on barriers are shown in Table 1. More than half of the participants (66%) were male and the mean age was 51.3 years (±7.3). The majority of the participants reported “lower secondary school” as the highest level of education (51%) and were suffering from low back pain for more than twelve months before inpatient rehabilitation (87%). Less than a quarter of the participants reported normal weight (22%), this means a body mass index between 18.5 and 25.0 kg/m^2^, and 58% of the participants were classified as physically active during leisure time at baseline. Six months after rehabilitation, the mean pain intensity was 3.6 (±1.3) on a scale from 1 to 6. The opinion of the participants in regard to fear of pain, which means the individual's assumption of a negative influence of physical activity on low back pain, could be graded as “not sure” (2.8 (±1.9) on a scale from 0 to 6) and 77% of the participants were classified active at six-month follow-up. Overall, no differences were observed in subject characteristics among randomization groups of the main study (results not shown).

### 3.2. Perceived Barriers of Inactive Participants

The evaluation of barriers on physical activity was restricted to the 44 participants reporting to be physically inactive during leisure time (0 MET-min/week) at six-month follow-up (*T*1).

**Figure 1:**
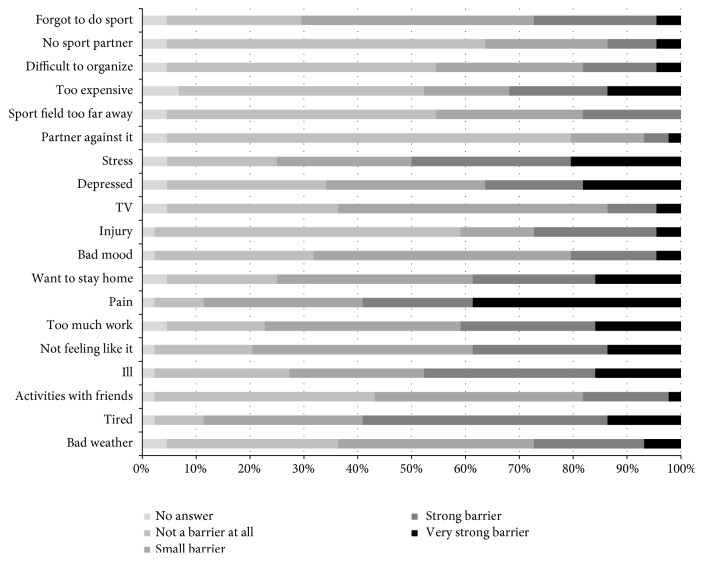
Perceived barriers of inactive participants at six-month follow-up (multiple answers possible).

More than half of the patients reported the internal barriers “being tired” (59%), “pain” (59%), and “stress (50%) as a strong or even very strong barrier to physical activity. Further major barriers were “being ill” (49%) and “too much work” (41%). From all barriers assessed, having a “partner not supporting” physical activity and sports (7%) and “watching” TV (14%) were reported least frequently (Figure 1).

### 3.3. Associations with Physical Activity following Rehabilitation

Table 2 shows frequencies for perceived barriers in inactive and active participants. Active and inactive patients showed statistically significant differences with regard to nine barriers, therefrom six internal barriers (“being tired” (*p* value < 0.001), “not feeling like it” (*p* value < 0.001), “want to stay home” (*p* value < 0.001), feeling “stressed” (*p* value = 0.014), being “depressed” (*p* value = 0.006), and “forgot to do sports” (*p* value < 0.001)) and three external barriers (“sport field too far away” (*p* value = 0.019), “too expensive” (*p* value = 0.004), and “bad weather” (*p* value = 0.005)), and they were reported proportionally more frequent in inactive patients. The highest proportional differences were in the barriers “want to stay home” (active: 10%; inactive: 39%) and “not feeling like it” (active: 14%; inactive: 39%).

The nine statistically significant barriers described above were included in a regression model (see Table 3). In combination with the sociodemographic and indication-related variables the model explained 51% of the variation (*R*^2^ = 0.51). Two variables were statistically significantly associated with leisure time activity: the odds of patients with a level of education higher than lower secondary school to be physically active were around five times higher than of patients with highest level of education “lower secondary school” (German: Hauptschule) (OR = 5.366; 95% confidence interval [1.563; 18.425]; *p* value = 0.008). Furthermore, the persuasion that physical activity increases low back pain was associated with statistically significantly higher odds of inactivity (OR = 0.612; 95% confidence interval [0.421; 0.889]; *p* value = 0.010). None of the nine barriers included in the regression model showed an association with physical activity in the adjusted regression model.

## 4. Discussion

The present analysis focused on barriers to physical activity among chronic low back pain patients participating in a specific program promoting physical activity following inpatient rehabilitation. Inactive and active participants differed significantly in nine of the 19 barriers assessed. The three main barriers of inactive participants were internal barriers (“being tired,” “pain,” and “stress”). The adjusted regression model showed that low level of education and fear of pain were significantly associated with physical inactivity. The barriers once included in the model did not remain statistically significant.

The association of education and leisure time physical activity or sports is widely accepted [28, 29]. Our results showed that a higher level of education was associated with a higher chance of being physically active during leisure time. Thereby, our results are in line with the literature showing that a low level of education is associated with a higher chance of being physically inactive in sports or during leisure time [29–31]. Hence, literature also shows, that, in contrast to leisure time activity, low level of education tends to be associated with higher workplace activity [32]. That the individual's fear that physical activity might increase low back pain was associated with a higher chance of physical inactivity during leisure time, again, is in line with other studies that were focusing on fear avoidance beliefs and chronic disability secondary to low back pain [33–35].

As the sustainable promotion of health-enhancing physical activity still is a big challenge [36, 37], the identification of target-group specific barriers to physical activity is of utmost importance. However, our adjusted regression model showed no statistically significant association of a specific barrier to physical activity. Barrier differences in active and inactive participants were confounded by education level and fear of pain. But the education level of low back pain patients cannot be changed by a physical activity promotion program and the change of fear of pain is a long lasting progress. Hence, for practical implications, taking a specific look at the barriers could give a clue about how to bypass these two major factors in future interventions. Beyond, developing physical activity promotion programs tailored to the educational level might be a promising approach on the long run.

Overall, the most common barriers mentioned in our study among low back pain patients reflect those found in other studies. In regard to external barriers, a study on barriers among persons suffering from chronic diseases across all chronic conditions also identified cost (corresponding to “too expensive” in our study) and travel time (corresponding to “sport facility too far away” in our study) as primary barriers [38]. Even though cost was identified as a barrier in several studies [39–41], in the light of the main study of the present evaluation that aimed at promoting physical activity in daily routine, it seems noticeable that cost was perceived as a barrier in our secondary analysis. With reference to internal factors lack of willpower (corresponding to “not feeling like it” in our study) [39, 40, 42–44] and existing physical ailments or chronic conditions [12, 42, 45] were identified as a barrier in several other studies. A noticeable aspect seems to be that in our study the barriers “pain” or “injury” showed a higher share in active participants. This leads to an interesting aspect: Probably the main difference between active and inactive participants might not be the specific barrier by itself but the ability to overcome it. While some internal barriers mentioned more frequently by inactive participants (“being tired”; “want to stay home”; “not feeling like it”; being “stressed” or “depressed”; “forgot to do sports”) seek further specific individual support, other barriers that showed a higher share in inactive participants (e.g., “bad weather,” “too expensive,” and “sport field too far away”) are rather external factors that are hardly to be overcome by direct support and need more complex approaches. This idea is supported by a narrative review on barriers to physical activity of patients with rheumatoid arthritis which showed that doing exercise did not influence the existence of barriers but showed that physically active patients appear to be more capable of overcoming them [46]. Veldhuijzen van Zanten et al. [46] concluded that the encouragement from health professionals and friends/family is facilitator for physical activity and exercise. Along the same lines are the results of a qualitative study on patients with chronic musculoskeletal pain suggesting that this patient group has a greater need for information and extra support to overcome existing barriers to physical activity [47].

In recent years, professional health coaching has become a promising approach to initiating behavioral changes and improving health [48, 49]. Thereby, health coaching is understood as a patient-centered education method aiming at motivating individuals to achieve health goals and improving self-management [50]. Again, this is an interesting aspect in light of the main study: in the main study, a “movement coach” was applied, supporting motivational and volitional aspects of physical activity promotion. Probably the focus on physical activity-related aspects was too narrow and psychosocial aspects as well as indication-related aspects and pain management should be more strongly integrated. As fear of pain is a modifiable factor, the relationship of pain and physical activity should be explicitly addressed during rehabilitation. In consequence, an interdisciplinary health coaching intervention conducted by a sport scientist and a psychologist might be a promising approach.

To our knowledge, this is the first study having investigated barriers to physical activity among low back pain patients who were participating in a trial promoting physical activity while in an inpatient rehabilitation clinic. Our study limitations include the recruitment from only one inpatient rehabilitation center which may limit generalizability of the results. Due to the main study, a second limitation certainly is that all participants in this secondary analysis were participating in a trial promoting physical activity voluntarily. In consequence, the results may not be generalized to patients who were less motivated to participate in the main study. Given the fact that we evaluated patients previously enrolled in a formal inpatient rehabilitation program the results may be less applicable to individuals suffering from low back pain who were not previously exposed to an intensive rehabilitation program. Furthermore, heterogeneity in the definition of physical activity and the corresponding classifications and in different questionnaires limits the comparison to other studies [51].

## 5. Conclusions

As the sustainable promotion of health-enhancing physical activity still is a big challenge [36, 37], the identification of target-group specific barriers to physical activity is of utmost importance. To develop application-oriented approaches in physical activity promotion, health professionals need profound information on the relationships between the barriers and sociodemographic and indication-related variables. Our results showed that there might be a need for especially supporting low back pain patients with a low level of education and fear of pain in overcoming internal and external barriers to physical activity.

## Figures and Tables

**Table 1:** Characteristics of the sample.

	Sample
*Baseline (T0)*	
*Age* (years) (*n* = 190) [mean (SD)]	51.3 (±7.3)
*Gender: *men (*n* = 192) [*n*; %]	126 (66%)
*Body mass index: normal weight* (*n* = 179) [*n*; %]	40 (22%)
*Highest level of education “lower secondary school”* (*n* = 188) [*n*; %]	100 (52%)
*Duration of low back pain at baseline > 12 months* (*n* = 187) [*n*; %]	182 (87%)
*Active (>0 MET-min leisure time physical activity/week)* (*n* = 192) [*n*; %]	111 (58%)
*Intervention group* (*n* = 192) [*n*; %]	92 (48%)

*Six-month follow-up (T1)*	
*Intensity of pain during the last four weeks at six-month follow-up (min: 1; max.: 6)* (*n* = 188) [mean (SD)]	3.6 (±1.3)
*Active following rehabilitation (>0 MET-min leisure time physical activity/week)* (*n* = 186) [*n*; %]	148 (77%)
*By physical activity my back pain becomes more intense (0: not at all; 6: exactly)* (*n* = 183) [mean (SD)]	2.8 (±1.9)

**Table 2:** Perceived barriers of inactive and active participants at six-month follow-up (dichotomized).

Perceived barrier (“very strong barrier” or “strong barrier”)	Inactive (*n* = 44) *n* (%)	Active (*n* = 148) *n* (%)	*p* value^1^
Bad weather	12 (27%)	16 (11%)	**0.005** ^**∗**^
Tired	26 (59%)	27 (25%)	**<0.001** ^**∗**^
Activities with friends	8 (18%)	19 (13%)	0.376
Being ill	21 (48%)	85 (58%)	0.276
Not feeling like it	17 (39%)	20 (14%)	**<0.001** ^**∗**^
Too much work	18 (41%)	49 (33%)	0.281
Pain	26 (59%)	64 (43%)	0.060
Want to stay home	17 (39%)	15 (10%)	**<0.001** ^**∗**^
Bad mood	9 (21%)	21 (14%)	0.302
Injury	12 (27%)	58 (39%)	0.125
TV	6 (14%)	10 (7%)	0.132
Depressed	16 (36%)	26 (18%)	**0.006** ^**∗**^
Stress	22 (50%)	46 (31%)	**0.014** ^**∗**^
Partner against it	3 (7%)	4 (3%)	0.202
Sport field too far away	8 (18%)	10 (7%)	**0.019** ^**∗**^
Too expensive	14 (32%)	21 (14%)	**0.004** ^**∗**^
Difficult to organize	8 (18%)	14 (10%)	0.100
No sport partner	6 (14%)	10 (7%)	0.132
Forgot to do sport	12 (27%)	8 (5%)	**<0.001** ^**∗**^

^1^Pearson chi squared; ^*∗*^*p value* < 0.05.

**Table 3:** Associations with physical activity.

*N* = 139	Beta	SE (*β*)	Sig.	OR	95%-CI
*Age* (years)	0.021	0.040	0.610	1.021	[0.943; 1.105]
*Gender:* men versus women	0.373	0.603	0.536	1.452	[0.445; 4.736]
*Body mass index:* “overweight or obese” versus “normal weight”	0.984	0.647	0.128	2.675	[0.753; 9.502]
*Highest level of education:* “higher than lower secondary school” versus “lower secondary school”	1.680	0.629	**0.008** ^*∗*^	5.366	[1.563; 18.425]
*Duration of LBP at baseline:* “≤12 months” versus “>12 months”	−1.357	1.094	0.215	0.258	[0.030; 2.196]
*Intensity of pain during the last four weeks at six-month follow-up* (min: 1; max.: 6)	−0.096	0.248	0.699	0.909	[0.559; 1.477]
*More back pain by physical activity* (0: not at all; 6: exactly)	−0.491	0.191	**0.010** ^*∗*^	0.612	[0.421; 0.889]
*Baseline leisure time physical activity:* “active” versus “inactive”	0.637	0.602	0.290	1.891	[0.581; 6.151]
*Study group:* “control group” versus “intervention group”	−0.789	0.564	0.162	0.454	[0.150; 1.373]
*Barrier: bad weather* “barrier” versus “no barrier”	−0.358	0.707	0.612	0.699	[0.175; 2.792]
*Barrier: being tired* “barrier” versus “no barrier”	−1.207	0.633	0.056	0.299	[0.087; 1.034]
*Not feeling like it* “barrier” versus “no barrier”	−0.648	0.670	0.333	0.523	[0.141; 1.944]
*Want to stay home* “barrier” versus “no barrier”	−1.075	0.706	0.128	0.341	[0.086; 1.362]
*Depressed* “barrier” versus “no barrier”	0.071	0.626	0.909	1.074	[0.315; 3.663]
*Stress* “barrier” versus “no barrier”	−0.056	0.617	0.928	0.945	[0.282; 3.166]
*Sport facility too far away* “barrier” versus “no barrier”	−0.374	0.929	0.688	0.688	[0.111; 4.255]
*Too expensive* “barrier” versus “no barrier”	−0.592	0.670	0.377	0.553	[0.149; 2.055]
*Forgot to do sport* “barrier” versus “no barrier”	−0.016	0.852	0.985	0.985	[0.185; 5.229]

Dependent variable: leisure time activity following rehabilitation (“active”: >0 MET-min/week versus “inactive”: 0 MET-min/week); ^*∗*^*p* value < 0.05; *R*^2^ = .051; variables not included. Barriers: activities with friends; pain; being ill; too much work; injury; TV; partner against it; difficult to organize; no sport partner. Odds ratios are adjusted for socioeconomic status variables and intervention group.
